# An Implicit Theories of Personality Intervention Reduces Adolescent Aggression in Response to Victimization and Exclusion

**DOI:** 10.1111/cdev.12003

**Published:** 2012-10-25

**Authors:** David Scott Yeager, Kali H Trzesniewski, Carol S Dweck

**Affiliations:** University of Texas at Austin; University of CaliforniaDavis; Stanford University

## Abstract

Adolescents are often resistant to interventions that reduce aggression in children. At the same time, they are developing stronger beliefs in the fixed nature of personal characteristics, particularly aggression. The present intervention addressed these beliefs. A randomized field experiment with a diverse sample of Grades 9 and 10 students (ages 14–16, *n* = 230) tested the impact of a 6-session intervention that taught an incremental theory (a belief in the potential for personal change). Compared to no-treatment and coping skills control groups, the incremental theory group behaved significantly less aggressively and more prosocially 1 month postintervention and exhibited fewer conduct problems 3 months postintervention. The incremental theory and the coping skills interventions also eliminated the association between peer victimization and depressive symptoms.

Adolescent aggression extracts a staggering cost in human suffering. Apart from the human toll, the direct and indirect economic costs exceed $158 billion per year in the United States alone (Children's Safety Network Economics & Data Analysis Resource Center's, 2008). Although some aggression may be unprovoked, many aggressive behaviors are responses to provocations, such as peer victimization or exclusion (Olweus, 1993; Ostrov, 2010; Reijntjes et al., 2010). Unfortunately, many preventative interventions that are effective for reducing aggression or conduct problems in younger children have yielded inconsistent findings for adolescent populations (Fossum, Handegård, Martinussen, & Mørch, 2008; Merrell, Gueldner, Ross, & Isava, 2008; Smith, Schneider, Smith, & Ananiadou, 2004; Vreeman & Carroll, 2007; Wilson & Lipsey, 2007)—the age when aggression becomes increasingly violent (Cairns & Cairns, 1994). Thus, it is essential to increase our understanding of the causes of adolescent aggression in response to victimization or exclusion, and to explore developmentally tailored interventions that can stem such aggression. The present research tests such an intervention.

We focus on implicit theories of personality—or beliefs about the potential to change personal characteristics—as a target for a new social-cognitive intervention. Adolescents' implicit theories of personality create a framework for their interpretations of setbacks, and have been shown to shape their vengeful and punitive versus prosocial and resilient reactions to conflicts, social failures, or a peer's wrongdoing (e.g., Yeager & Miu, 2011; Yeager, Trzesniewski, Tirri, Nokelainen, & Dweck, 2011; for related findings with children, see Erdley, Cain, Loomis, Dumas-Hines, & Dweck, 1997; Erdley & Dweck, 1993; Giles & Heyman, 2003; Rudolph, 2010; see also Dweck, 1999). Specifically, some adolescents hold more of an *entity theory* of personality, which is the belief that people's traits are fixed (Yeager et al., 2011). They believe that people who are “bullies” or “victims,” “winners” or “losers,” cannot change. From this perspective, victimization or exclusion may be seen as done by and to people who cannot change—for example, by a “bully” to someone who is considered a “loser.” Under these conditions, harming the transgressor may seem satisfying. On the other hand, some adolescents hold more of an *incremental theory* of personality, believing that people have the capacity for change (Yeager et al., 2011). Seen from this perspective, victimization may be thought of as done by and to people who can change over time. This may reduce aggressive retaliation by allowing students to see their future as more hopeful and by creating a greater desire to understand or perhaps influence transgressors.

## Implicit Theories Predict Desire for Vengeance

With experience, people form foundational beliefs about causal processes in their social worlds, and these beliefs can shape their interpretations of events in their environment as well as their patterns of behavior (Dweck, 2008). Theories of aggressive behavior have suggested that such beliefs, once formed, can motivate hostile or resilient reactions to victimization or exclusion (Bushman & Huesmann, 2010; Crick & Dodge, 1994; Dodge, Coie, & Lynam, 2006). As noted, we contend that one set of beliefs that can impact the motivation to retaliate is a belief about people's potential to change—that is, their implicit theories of personality.

Research has demonstrated that implicit theories of personality predict the degree to which adolescents wish to respond vengefully to victimization. In recent work by Yeager et al. (2011), conducted with diverse samples in the United States and Finland, adolescents who held more of an entity theory—believing that people cannot change—also harbored a greater desire to “get back at” peers who had insulted or excluded them, and to dream of ways to “give them what they deserved” (Yeager et al., 2011, Study 1). Similar results were obtained when adolescents responded to a hypothetical conflict involving direct victimization in school (Yeager et al., 2011, Study 2). Interestingly, there were no differences across nations or across ethnic or racial groups in the impact of an entity theory on the desire for vengeance, suggesting that implicit theories of personality may influence the motivation to respond aggressively in many contexts.

There is already preliminary evidence that implicit theories of personality can be changed, leading to changes in the desire for vengeance. In a short experiment, Yeager et al. (2011, Study 3) used an established method to temporarily shift adolescents' mindsets toward more of an incremental view. Half of the participants read a brief story about a student who was a victim of bullying in school and learned from peers and adults that people's characteristics are malleable and not fixed. Adolescents who received this incremental theory message, compared to those who read the same scenario without it, were significantly less likely to later say that if they were the victim they would choose aggression as a response to the bullies. Instead, they were more likely to choose prosocial solutions, such as educating the transgressors or explaining to them the effects of their actions (see also Yeager & Miu, 2011).

Based on this past research, we hypothesized that an intervention that taught adolescents an incremental theory of personality and how to apply it to interpersonal situations would reduce aggressive retaliation. Notably, no such intervention has been conducted previously. Yeager et al.'s (2011) experiment used a hypothetical scenario, measured only self-reported desire to take revenge, and did not involve a longitudinal follow-up. Therefore, the present research advances previous implicit theories work by (a) using a more comprehensive incremental theory message, (b) systematically teaching adolescents how to apply this message to peer conflicts, (c) comparing this intervention with a parallel intervention that taught coping skills but did not teach that people can change, and (d) assessing behavior 1 and 3 months postintervention.

It is important to note that the ultimate goal of research in this area is to find ways to reduce bullying and victimization in general, and not only to help students cope with instances of victimization. However, as long as victimization (or perceived victimization) exists, it is important for adolescents to respond in nonviolent ways. Moreover, to the extent that victims find other victims to bully (e.g., Reijntjes et al., 2010), reducing aggressive responses may also serve to reduce bullying.

## The Need for Developmentally Appropriate Methods for Reducing Adolescent Aggression

With development, children and adolescents seem to believe that it is less possible to change traits such as meanness and aggression. For instance, Lockhart, Chang, and Story (2002) examined young children's and older children's beliefs about their peers' traits. When the children evaluated a peer who was described as being mean but wanting to become nicer, older children were more pessimistic about the peer's prospects for change as compared to younger children (see also Hymel, Wagner, & Butler, 1990). In line with this, Diesendruck and colleagues (Birnbaum, Deeb, Segall, Ben-Eliyahu, & Diesendruck, 2010; Diesendruck & haLevi, 2006), who examined kindergarteners, sixth graders, and adults, showed that traits such as niceness or shyness—as opposed to group membership—became increasingly relied on from childhood to adulthood when making social judgments about a peer. Most directly relevant to the present study, Killen, Kelly, Richardson, and Jampol (2010) found that high school students, compared to middle school students, were more likely to think of aggression as a stable trait. Adolescents judged an ambiguous event in which it was unclear whether a peer had pushed another student down or offered to help the student stand up, and researchers varied whether the peer was described as having a history of aggression or not. In comparison with middle school students, high school adolescents thought it was fairer to accuse the target student of intentionally pushing the other peer if the student had previously been caught pushing someone. Overall, then, past research is suggestive that fixed beliefs about the traits of aggressive peers may be especially likely to play a role in a desire to retaliate aggressively.

In light of these findings, one might expect that when interventions do not explicitly address beliefs about the fixedness of people's characteristics, then they may be less effective in reducing retaliatory aggression for adolescents than they would be for younger children. In fact, many evaluations of universal preventative interventions are in line with this expectation. Specifically, universal interventions that taught coping skills or that attempted to change school culture have had consistent success in reducing levels of aggression with children, whereas similar interventions have yielded more inconsistent results with adolescents, with many showing null or even negative effects, despite evidence that these programs were implemented faithfully (e.g., Silvia et al., 2011; for extensive narrative reviews and meta-analyses, see Fossum et al., 2008; Merrell et al., 2008; Smith et al., 2004; Vreeman & Carroll, 2007; Wilson & Lipsey, 2007). As one example, Karna et al. (2011) delivered a universal antibullying intervention in 888 schools to roughly 150,000 students across the entire nation of Finland. This intervention strongly and significantly decreased bullying among children 8–13 years old but had no significant effect among adolescents 14–16 years old. Of course, this could be because aggression does in fact become a more stable personality characteristic with age, but it could also be that adolescent aggression is difficult to reduce unless beliefs about the malleability of personal characteristics are explicitly addressed.

## Relation to Previous Implicit Theories Interventions

One reason to expect that an implicit-theories-of-personality intervention might change behavior is that implicit-theories interventions have been conducted previously with adolescents in the academic domain, showing effects on behavior in the classroom. Blackwell, Trzesniewski, and Dweck (2007, Study 2) conducted an eight-session intervention in which the experimental group learned an incremental theory of intelligence (the idea that intelligence can be developed) in two sessions along with six sessions of study skills. For example, students learned that every time they stretched themselves to learn something new, the brain formed new connections and that, in this way, over time they could become smarter. Blackwell et al. (2007) compared the incremental group with a control group that received eight sessions of study skills. Students in the study-skills control group continued the normative decline in math grades, but students who learned the incremental theory plus study skills reversed this trend and showed a rebound in their grades (see also Aronson, Fried, & Good, 2002; Good, Aronson, & Inzlicht, 2003).

The present experiment was similar to Blackwell et al.'s (2007) in that it compared an incremental theory intervention with a control group that learned only extensive domain-relevant skills (in this case, coping skills). Blackwell et al. (2007) showed that simply learning skills alone was not enough to create a new pattern of behavior; rather, adolescents needed the motivation to put the skills into practice. The present research capitalized on the findings of Blackwell et al. (2007) but was also unique in that it focused on a different implicit theory—about the kind of person someone is—with the goal of reducing aggression. We hypothesized that teaching only social coping skills would not reduce aggression, while teaching the incremental theory of personality would provide students with a new framework for understanding the social world, thereby helping them put their skills into practice.

## Peer Victimization and Depression

Research has found that peer-victimized youth exhibit more depressive symptoms (e.g., Klomek, Marrocco, Kleinman, Schonfeld, & Gould, 2007; Rudolph, 2010) and that there are similarities between the social-cognitive processes leading to aggressive and depressive symptoms following victimization or exclusion (e.g., Graham, Bellmore, & Mize, 2006; Hawker & Boulton, 2000; Rudolph, 2010). One theory is that the same implicit beliefs about aggressive peers' fixed traits (e.g., “He's a bad person”) may be applied to one's own personal deficiencies (e.g., “I'm a loser”) and thereby produce a vulnerability that can result in depressive symptoms. That is, an entity theory may lead to the conclusion that one's own social labels and difficulties cannot be improved, inducing hopelessness (e.g., Heyman, Dweck, & Cain, 1992). It is therefore possible that an incremental theory intervention that teaches the idea that “bullies” and “victims” can change might also reduce depression as well as aggression. Indeed, in a correlational study, Rudolph (2010) found that peer-victimized fifth-grade students with more of an entity theory reported more depressive *and* aggressive symptoms in school than victimized students with more of an incremental theory. The present experiment will extend this correlational research by assessing the causal effect of an incremental theory intervention on both aggression and depression among adolescents who report that they were or were not peer victimized.

## The Present Research

On the basis of these past theories and findings, we designed an intervention to teach high school students how to apply an incremental theory of personality in their daily lives, especially following incidents of victimization or exclusion. Students attending a diverse, low-income public high school with substantial levels of conflict were randomly assigned to a six-session incremental theory intervention or to one of two control groups. One control group received six sessions of instruction in social-emotional coping skills. These sessions taught students new strategies for thinking positively following conflicts or setbacks and new ways to resolve conflicts productively. Similar skill-building interventions are commonly administered to adolescents (e.g., Frydenberg, 2010; Silvia et al., 2011) and are frequently effective at reducing aggression among children (Wilson & Lipsey, 2007). A second control group received no treatment.

We measured effects 2 weeks, 1 month, and 3 months postintervention, testing the hypotheses that an incremental theory intervention would:

1. reduce retaliatory aggression and increase prosocial behavior when students were exposed to a standardized incident of peer exclusion (i.e., when everyone experienced the same victimization by peers);
2. reduce conduct problems in school as observed by teachers, primarily among those who reported being victimized by their peers; and
3. reduce depressive symptoms, primarily among those who reported being victimized by their peers.

## Method

### Sample

#### School recruitment

The participating school was a medium-to-large-sized high school in the San Francisco Bay Area. The school was randomly selected from 20 schools in the area that had more than 1,000 students and that were near the mean for the area in terms of the proportion of students who were (a) English language learners; (b) White, non-Latino; (c) “proficient” on a standardized English test; and (d) eligible for free or reduced-price lunch. We limited our pool to relatively large schools to minimize contamination across experimental conditions. The selected school administered a climate survey, and on it 70% of students did not agree that students treat each other with respect, and 40% said that they did not feel safe from threats at school.

#### Participants

There were 246 ninth- and tenth-grade students in the study (14–16 years old). Sixteen students (evenly distributed across conditions) did not attend half or more of the sessions, or missed the final session (which included a critical activity), and were therefore excluded, leaving a final sample of 230 students. (Note that we also conducted separate analyses with the full sample of 246 students to calculate an intent-to-treat [ITT] effect of the intervention. We found that the conclusions about the effects of the incremental theory intervention on aggression were not different in the ITT sample.) Fifty-five percent were boys and 45% were girls, and 57% were Latino, 10% were Asian American, 9% were African American, 17% were White, non-Latino, and the rest indicated another race or ethnicity. Sixty-four percent received free or reduced-price lunch. Four percent of parents had a graduate degree, 18% had a college degree, 26% had some college education (but no degree), and the rest had a high school degree or less. Parental consent and student assent were obtained for each participant. All invited students assented to participate in the workshops and provide official data, but some students declined to participate in some of the data collection procedures (described next).

### Procedure

#### Overview

**Figure 1:**
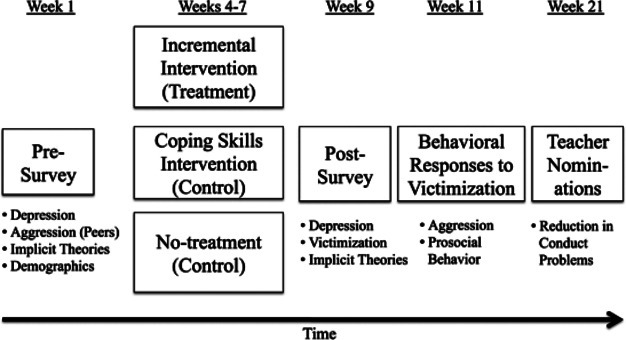
Design of the study.

An overview of the procedure is presented in Figure 1. Two weeks before the interventions began, a survey was administered during school hours and a similar postsurvey was administered 2 weeks postintervention. One month postintervention, a standardized task that yielded behavioral responses to peer victimization was administered, and at the end of the semester (3 months postintervention), teachers completed a survey in which they nominated students for reduced conduct problems. We also collected information from school records. To minimize any connection between the interventions and the dependent measures, different research assistants collected the measures during a different class than the one in which the workshops were delivered.

#### Random assignment

In partnership with the school, before the semester began, participating students were randomly assigned to one of nine different biology classes. Next, three of the nine classes of biology (with approximately 27 students in each section) were randomly assigned to each condition: the incremental theory group, the coping skills control group, or the no-treatment control group. Comparisons of the groups revealed that random assignment was effective with regard to all variables measured at baseline (see online Supporting Information). The incremental theory and coping skills interventions lasted 3 weeks and included six class sessions of about 50 min each, delivered in biology classes.

#### Facilitators

Two male and two female adult paid facilitators were recruited to conduct the interventions. Facilitators had 2–10 years of experience teaching or working with diverse adolescents from low-income areas, but had no previous specialized training in interventions to reduce aggression. Two teams were created—each with one male and one female (both females spoke Spanish fluently). Once the two teams were created, they were randomly assigned to administer either the incremental theory intervention or coping skills intervention. Follow-up tests showed that facilitators in both groups were indeed blind to hypotheses, and all of the facilitators independently guessed that their group was the treatment group. In addition, follow-up interviews with the school's science faculty confirmed that they did not know the content of the two workshops or the dependent variables in the study.

Both teams of facilitators received equal amounts of training time from the researcher (about 40 hr) and were highly enthusiastic about the treatment they administered. All facilitators learned about the brain, but only the incremental theory team learned how the core abilities of the brain could be changed and grown. The coping skills facilitators, but not the incremental theory facilitators, also read two books written by the author of the coping skills curriculum and discussed them with the researcher.

### Incremental and Coping Skills Workshops

#### Overview

The incremental theory and coping skills workshops were parallel in many ways. The activities students completed, the texts they read, and the lectures they heard were similar or identical for much of the workshops (see Table 1). The groups differed, however, in that the incremental theory intervention taught the idea that people have the potential for change, especially in the context of victimization or exclusion, but did not teach specific actions to take following social adversity. The coping skills intervention, on the other hand, explicitly taught skills for thinking positively and coping productively in the face of victimization or exclusion, but did not explicitly target students' construals of the social world. Critically, surveys and quizzes showed that both of the workshops were equally enjoyable and taught an equal amount of knowledge (see online Supporting Information).

**Table 1:** Overview of Incremental Theory and Coping Skills Interventions

Session	Incremental theory (treatment)	Coping skills (control)
1 and 2	*Message*: Neuroanatomy + How the brain changes with learning.	*Message*: Neuroanatomy + How the brain can be used for learning.
	*Activities*: Team-building; lectures; complete “brain challenge” worksheets.	*Activities*: Team-building; lectures; complete “brain challenge” worksheets.
3 and 4	*Message*: People's personalities live partially in their brains, and brains can be changed.	*Message*: Use productive coping strategies and avoid unproductive coping strategies.
	*Activities*: Lectures; practice using the incremental theory in response to hypothetical rejections or interpersonal conflicts.	*Activity*: Lectures; practice using the coping strategies in response to hypothetical rejections or interpersonal conflicts.
5 and 6	*Message*: People have many motivations for their actions besides their personalities (like thoughts and feelings), and some of these can also change.	*Message*: Think positively: avoid “thought distortions” like “overgeneralization” or “all or nothing thinking” and this will help keep your life in balance.
	*Activities*: Write and perform skits using the incremental message in response to rejection or conflict; small-group discussions; final writing assignments.	*Activities*: Write and perform skits using coping skills in response to rejection or conflict; small-group discussions; final writing assignments.

#### Designing the incremental theory message

On the basis of extensive interviews and pilot testing with youth similar to those targeted by our actual intervention, we developed several themes that guided how an incremental theory was taught in our intervention. First, based on what pilot students found most persuasive and realistic, we argued that changing personality is not easy—it is hard, can take a long time, and often requires a great deal of help from others—but it is always possible. Second, consistent with past research (Yeager et al., 2011), we emphasized an overall mindset that people can change, both aggressive students and students who were picked on or left out—that is, both others and oneself. Third, we discussed various mechanisms of change generated by the pilot students, including maturity, motivation, situations (both moment to moment and over time), help from others, change-inducing experiences that open your eyes to the effects of your behaviors, or pressure from parents. To this list, we added the idea of reorganized brain pathways resulting from learning and from new patterns of behavior. Finally, throughout the intervention, we quoted extensively from the arguments pilot students generated in support of an incremental theory.

#### Methods to increase the impact of the incremental theory message

In line with previous successful interventions (Aronson et al., 2002; Walton & Cohen, 2007; see Yeager & Walton, 2011), we avoided making students feel that they were in need of “an intervention.” Instead, in both the treatment and control groups, we framed the activities as a way for them to mentor next year's ninth-grade students (who were later, in fact, given the mentoring letters; cf. Aronson, 1999). We also communicated *descriptive norms* (Cialdini, 2003) that, in the incremental group, emphasized that *students like you* successfully used an incremental theory and did not use an entity theory after they had setbacks in life and in school (cf. Walton & Cohen, 2007). Finally, our intervention was a universal intervention delivered generally, and not to a subset of aggressive youth, following recommendations designed to prevent the stigmatization or “deviancy training” that can result from interventions that form new peer groups of at-risk youth (Dishion & Tipsord, 2011; Dodge, Dishion, & Lansford, 2006).

### Content of the Incremental Theory Intervention

The six incremental theory intervention sessions were divided into three segments of two sessions each. These segments had two aims: to teach the science behind the incremental theory and to provide opportunities to practice the theory in the context of social conflicts they experienced or imagined. The intervention did not tell students specific ways to resolve problems or explicitly teach new skills for interacting with their peers—that is, it did not make any direct statements about the use or avoidance of aggression to solve problems. Instead, it sought to change students' construals of themselves and others following victimization or exclusion. The three segments are described next and summarized in Table 1.

#### Sessions 1 and 2

The objective of the first two sessions was to teach about neurons, and to introduce the idea that the brain is malleable and can be changed with effort and experience (cf. Blackwell et al., 2007). We started our intervention by teaching about the malleability of intelligence because, in pilot studies, students found this material to be a helpful analogy for understanding how other traits could change through effort, experience, and help from others.

#### Sessions 3 and 4

Days 3 and 4 were designed to provide the bulk of the material teaching an incremental theory of personality. The sessions began with a discussion of famous people who encountered and overcame social rejection, so that students could discuss role models for whom social adversity did not last forever. Next, the workshop transitioned to an explanation of the mechanisms that support the incremental view of personality. Specifically, the facilitators told students:

Scientists have discovered that people do things mainly because of the thoughts and feelings that they have—thoughts and feelings that live in the brain and that can be changed. When you have a thought or a feeling, the pathways in your brain send signals to other parts of your brain that lead you to do one thing or another. … By changing their brain's pathways or their thoughts and feelings, people can actually change and improve how they behave after challenges and setbacks. So it's not that some people are “rejects” or that other people are “bad.” Everyone's brain is a “work in progress.”

Students were next presented with scientific evidence explaining that people's habits live in the brain and can be changed. For example, they were shown images of fMRIs from patients who had relocalized various functions after brain traumas and were told about the impact this had on their behavior. Additionally, facilitators presented the results of longitudinal studies documenting changes in people's traits (like aggression or peer rejection) over time. Students completed targeted writing assignments in which they practiced applying the incremental theory to reconstrue a variety of social adversities, such as exclusion, rejection, and aggression.

#### Sessions 5 and 6

These sessions were designed to cement the incremental theory in students' minds, to practice it in additional scenarios, and to correct misconceptions. For example, a group of boys and a group of girls each separately created a skit in which they acted out a conflict with aggressive students in school or an experience of social rejection and used an incremental theory in response to it. During these skits, students did not practice new ways to *solve* these problems, but instead focused on how to *think about* the problem through the lens of an incremental theory. Students also broke up into same-sex pairs and practiced presenting the incremental theory to each other. During this activity, one partner was an “alien from the planet of the entity theory,” whereas the other partner was responsible for explaining the incremental theory to the “alien,” who pretended to have never heard of the incremental theory before. In another activity, students again broke up into two small groups, and various misconceptions were corrected. Specifically, this conversation centered on the following questions: (a) Does a growth mindset mean that I will change into a completely different person from one day to the next? If I'm a moral person today, does the growth mindset mean that I could be an evil person tomorrow? (b) My family member has a bad habit—is it my job to change them by myself? and (c) If the people who pick on me or are aggressive toward me aren't “bad,” does that mean it's my fault for getting picked on? The facilitators discussed why the answer was “No” to each of these questions.

Finally, students were asked to write about “one time when you felt left out, rejected, or upset by an acquaintance at school” and then to “imagine that the same event you described happened to another student just like you” and say something to help the future ninth grader “understand that they can change and that the things that are happening to them could change.” As one typical example, a female participant in our study wrote:

Recently, people have been calling me mean names. The most common is “loner.” … Although I was very upset, I have gotten over it … the insults aren't going to last forever. As they mature and change they'll stop acting so foolish. … And I know that as I grow and get older I'll develop more friends.

### Content of the Coping Skills Intervention

Great effort, time, and resources went into making the control group intervention a highly enjoyable and informative experience that was similar to the incremental theory group. Most of the content was based on a popular coping skills curriculum that is widely used with high school students and that focuses on skills for coping with social adversity and on thinking positively (Frydenberg, 2010). As noted, this type of coping and social problem-solving training frequently reduces aggression in younger children and is often applied to older high school-aged adolescents. The full curriculum included up to 12 sessions, which was longer than our intervention period. Therefore, we administered only the content relevant to coping with social setbacks, and not the remaining content, which dealt with goal setting and academic skills.

To this content, other activities and materials were added to make these sessions more parallel to the treatment. As in the treatment group, the coping skills control group was told that they were learning the content to mentor next year's ninth-grade students. The messages communicated by each session of the coping skills intervention are summarized in Table 1.

#### Sessions 1 and 2

The majority of the content during these sessions was similar to the incremental theory intervention in order to increase the comparability of the two interventions. The same information about neuroanatomy was presented; only the message about the brain's ability to change was omitted and replaced with information about improving memory and performance (cf. the control group in Blackwell et al., 2007, Study 2). Students were taught how the brain can be used for learning rather than how the brain can be changed with learning. Although memory and brain function are not normally part of coping skills curricula, these activities were included in order to make the beginning of the workshop as similar to the incremental theory intervention as possible. Students also engaged in numerous team-building exercises with the dynamic facilitators that promoted camaraderie.

#### Sessions 3 and 4

Days 3 and 4 were designed to introduce the majority of the coping skills content. These sessions began with a discussion of famous people who encountered and overcame social rejection, and of what coping skills might help them overcome similar instances of social rejection. Next, the workshop transitioned to an explanation of the coping strategies that are helpful and not helpful for dealing with similar social adversities. Most of the strategies emphasized emotion-focused coping (changing one's emotional reactions to a conflict), including “focusing on the positive” and “avoiding self-blame.” However, there was also a strong emphasis on problem-focused coping (finding ways to prevent and resolve conflicts). In line with this, there were exercises on three occasions during which students read scenarios of social conflicts and were asked to generate and then describe ways of solving the problem. Overall, students practiced applying the helpful coping strategies (and not using the unhelpful coping strategies) in many of the same peer conflict scenarios as used in the incremental theory intervention.

#### Sessions 5 and 6

These sessions were designed to reinforce and extend the coping skills content, and to practice it in scenarios of peer conflict that could potentially result in aggression. During these sessions, students were given a list of thought distortions to avoid, such as “all or nothing thinking,” “catastrophizing,” or “overgeneralization.” They were told how these distortions, when they are used after adversity, could lead to low self-esteem and anxiety and could get in the way of having success in life. They were given scientific evidence that people are happiest when their lives are in equilibrium due to the use of positive thinking, which can help one stay in a balanced state. Through skits and targeted writing assignments, students practiced using positive thoughts and behaviors and not using thought distortions after a series of hypothetical social conflicts (such as “A friend of yours is having a party this weekend and didn't invite you”).

Finally, students completed a self-persuasion writing assignment that emphasized using positive coping skills and avoiding negative coping skills. The language and formatting of this assignment were nearly identical to that in the incremental theory group. For instance, participants were also asked to “Describe one time when you felt left out, rejected, or upset by an acquaintance at school” and then to describe “productive coping strategies you used” and “thought distortions you did not use” in order to “teach other 9th graders about successful coping strategies.” The following is a typical example of a participant's response to the assignment:

There was one time where I felt rejected, because I wanted to play in a football game, and nobody picked me to be on their team. … I just sat down alone by myself. … I solved the problem by focusing on the positive, and it helped a lot.

### Measures

#### Survey sample

Two hundred participants (87% of the full sample) completed a presurvey and 171 (74% of the full sample) provided data for the postsurvey. The number of missing surveys did not vary by condition, and participants missing postsurvey data were no different from those with postsurvey data on any variable, except (not surprisingly) for absences or lateness. Four students who began the postsurvey did not complete some of the measures, leaving a sample of 167 for analysis of those data. In order to include the participants who did not complete the presurvey in analyses of the outcome data and avoid the bias that often results from list-wise deletion, missing values were estimated using the multiple imputation software Amelia II (King, Honaker, Joseph, & Scheve, 2001). Specifically, following best practices for minimizing bias in estimating missing values, we used the Amelia package in R (R Development Core Team, 2008) to create 50 imputation data sets that were subsequently combined in the analysis phase using the Zelig package (Imai, King, & Lau, 2007). This is a conservative approach that uses the available data without artificially inflating statistical power. No values for postintervention survey responses were imputed.

#### Official school records

Student sex, grade level, absences in core subjects (science, math, English, and social studies), and lateness in core subjects were obtained from official school records. We also obtained baseline grade point average in core subjects and suspensions for fighting or defiance to test for preintervention differences.

### Manipulation Checks and Control Variables

#### Adolescents' implicit theories of personality

To assess whether the intervention changed theories of personality, six items measuring adolescents' implicit theories of personality (Yeager et al., 2011) were administered on the pre- and postsurveys. Students were asked to agree or disagree with statements about whether people who are “bullies,” “victims,” “winners,” or “losers” can change (e.g., “Bullies and victims are types of people that really can't be changed”; 1 = *strongly disagree*, 6 = *strongly agree*). The items were averaged and combined into a single scale, with higher values corresponding to more of an entity theory of personality (α_pre_ = .76, *M*_pre_ = 2.96, *SD*_pre_ = 1.01; α_post_ = .84, *M*_post_ = 2.78, *SD*_post_ = 1.07; range = 1–6).

#### Baseline levels of aggression (peer nominations)

Using a measure developed by Thomaes, Bushman, Orobio de Castro, Cohen, and Denissen (2009), students circled the names of students in the study whom they had seen enacting physical, verbal, and relational aggression in the past week. Following Thomaes et al. (2009), nominations for each of these three categories were summed to create a composite (*M* = 3.86, *SD* = 2.20, range = 0–9).

#### Demographics

On the presurvey, participants were asked to indicate their race or ethnicity. Missing values for this measure were not imputed.

### Behavioral Responses to Standardized Peer Victimization or Exclusion

#### Sample

For the assessment of behavioral responses to peer exclusion or victimization, 150 of the 246 participants (balanced for age, sex, and experimental condition) were selected to participate, and 111 (74%) came to the school's computer laboratory on the day of the study and completed the task. There were no significant differences between the participating and nonparticipating sample (or across experimental conditions) on any baseline variable we measured. A subsample of students was used in order to complete this assessment in 1 day, so as to prevent participants from informing each other about the tasks. Participants who had not attended enough sessions (*n* = 6), who did not comply with the protocol (*n* = 7), or who indicated they were suspicious (*n* = 6) were dropped from the analyses, yielding a final sample of *n =* 92.

#### Overview of procedure

We measured adolescents' behavioral responses to victimization or exclusion 4 weeks after the end of the workshops. Five adult experimenters, different from the workshop leaders and blind to participants' intervention condition, conducted the sessions. This task had several parts. First, students played a computer game during which they experienced peer exclusion or victimization. Next, they had the opportunity to retaliate and to act prosocially. Then, they played the computer game again, and this time they were included by their peers. Last, students were thanked, debriefed, and given a $10 gift card for participating. The procedures are summarized next; greater detail can be found in the online Supporting Information.

#### Provocation

First, we followed a standard procedure to implement the provocation: peer exclusion via Cyberball (Williams & Jarvis, 2006). In this game, students think they are playing catch online with two other players (in fact the other players are controlled by the computer program). After being thrown the ball twice, participants are not thrown the ball again for the remainder of the game, which in past research has led to feelings of exclusion (Williams & Jarvis, 2006). As with any procedure that has the potential to evoke negative feelings, we were extremely concerned about ensuring students' well-being. Thus, the experience of exclusion was brief, mild, quickly followed by an experience of inclusion, and thoroughly explained to students during debriefing.

#### Aggression

Next, students were given an opportunity to retaliate aggressively by allocating hot sauce to one of the peers who excluded them during the game of Cyberball (Lieberman, Solomon, Greenberg, & McGregor, 1999). This activity was presented as a “taste-testing” activity in which their partner had to consume the entire amount of food allocated to them, and participants were told that, to save time, one of the two peers they just played Cyberball with would be their partner. They also learned that this peer did not like spicy foods. The measure of aggression was the number of grams of hot sauce allocated to the partner from the Cyberball game, knowing the partner would not like eating it (*M* grams of hot sauce = 34.36, *SD* = 32.60, range = 0.425–100 g; higher values corresponded to more aggression). Importantly, a meta-analysis of dozens of similar experiments found that such controlled procedures, although relatively benign, can be relied on to produce results that are valid for understanding real-world aggression (Anderson & Bushman, 1997). Indeed, in the present sample, students who were suspended for fighting allocated twice as much hot sauce (see online Supporting Information). As with the exclusion procedure, we were very sensitive to the issue of students thinking that they had caused discomfort to another person, and so during debriefing students were assured that the peer did not actually consume the hot sauce.

#### Prosocial behavior

Last, the procedure provided an opportunity for students to behave prosocially. Students were given a blank piece of paper and were told they could write a note to accompany the hot sauce, and that they could say whatever they would like. Notes were categorized by two independent coders blind to condition, using a codebook in which prosocial notes were defined as including friendly warnings about the spiciness of the food, apologies for having to assign spicy food, or friendly comments in general (1 = *prosocial*, 0 = *not prosocial*; Krippendorff's α = .88; percent writing prosocial notes = 23.33%). A typical example of a prosocial note was “I tried to put only a little bit of the hot sauce as I could because you circled you disliked it. So I hope it is not too much for you.” A typical example of a not-prosocial note was “I gave you a lot because you don't like spicy!! (and because you didn't share the ball)” (see online Supporting Information). During debriefing, students were told no one received their notes.

#### Teacher nominations for reductions in conduct problems

Three months after the end of the intervention, 16 ninth- and tenth-grade teachers were asked to report on reductions in conduct problems. Previous research has found that peer victimization and exclusion can give rise to a constellation of conduct problems, including both aggression and the related construct of acting out (i.e., classroom delinquency and decreased attentiveness in class; Hanish & Guerra, 2002). Therefore, our measure of conduct problems included both sets of behaviors.

Teachers, blind to experimental condition and hypotheses, completed an online survey in which they first indicated, from a list of students, those who had exhibited “clear and notable improvements in behavior in class” including “following directions, being respectful, and being kind or friendly” in the past 3 months. Then, on a separate screen, they indicated students who improved in “aggressive behavior toward students,” including “making fun of other students, hitting, slapping, pushing, threatening, excluding, spreading rumors, or insulting.” Conceptually replicating previous research (Hanish & Guerra, 2002), teacher nominations for reductions in aggression were significantly related to nominations for improvement in classroom misbehavior, tetrachoric *r* = .66, *p* < .05. These two scores were therefore averaged for each student to create a composite measure (*M* = 0.37, *SD* = 0.50, range = 0–2).

### Posttest Survey (2 Weeks Postintervention)

#### Depressive symptoms

In order to gauge the impact of the interventions on adolescents' reported depression, the 10-item short form of the Children's Depression Inventory (CDI; Kovacs, 1992) was administered on both the pre- and postsurveys. Items (which were summed) asked which of three levels of a symptom described them best (e.g., 2 = *I feel like crying everyday,* 1 = *I feel like crying many days*, 0 = *I feel like crying once in a while*; α_pre_ = .82, *M*_pre_ = 2.61, *SD*_pre_ = 3.11, range = 0–17; α_post_ = .83, *M*_post_ = 2.51, *SD*_post_ = 3.34, range = 0–14).

#### Peer victimization

Based on past correlational findings (Rudolph, 2010), we predicted that the effect of implicit theories on depressive symptoms and conduct problems would be greatest among those who were victims of peer aggression during that period of time. Therefore, on the posttest survey, our measure (based on Thomaes et al., 2009) asked participants to indicate how frequently in the past few weeks they had experienced physical aggression (“Got kicked, pushed or hit”), verbal aggression (“Called names or had mean things said to you”), and relational aggression (“Had rumors or lies spread about you” and “Been excluded or left out by other students”), on a 5-point scale (1 = *never*, 5 = *all the time*). These four items were averaged into a single scale with acceptable internal consistency (α = .78). The resulting measure was highly nonnormal, Shapiro–Wilk *W* = .95, *Z* = 4.31, *p* < .00001, and so victimization status was dichotomized. Participants whose average score was close to *never* (i.e., ≤ 2) were considered “nonvictims” (54%) whereas those whose average score was > 2 were considered “recent victims” (46%). Victimization was measured postintervention so that we could more precisely relate students' behaviors and experiences to their recent reports of victimization. We found that levels of postintervention victimization were no different across conditions, χ^2^(2) = 3.14, *ns*, and that characteristics of victims did not vary by condition (i.e., the size of the relation with correlates of victimization status were not significantly different across conditions).

## Results

Here, we present the results of least squares (OLS) regressions (for analyses of all but one dependent variable) or logistic regressions (for analyses of prosocial notes) of the dependent measures of interest on a dummy variable indicating experimental condition (1 = *incremental theory*, 0 = *coping skills or no treatment*) and on covariates. The coping skills group did not differ from the no-treatment control group, except on one dependent measure; therefore, the two control groups were combined in all other analyses. Throughout, we use the *p* < .05 level to indicate statistical significance.

None of the results were significantly moderated by participant sex, grade level, or baseline peer nominations for aggressive behavior. However, to minimize error variance, we controlled for these three variables in all analyses. (Nearly identical results were obtained when we did not include these controls.) Further, no results were moderated by participant race or ethnicity, and there were no significant mean level differences across race and ethnicity for any of the main study variables. The covariate-adjusted means reported throughout the text were calculated using the Zelig package (Imai et al., 2007). This approach models random variation in both measurement error and effect sizes to produce estimates.

### Manipulation Check

As expected, 2 weeks postintervention, students in the incremental theory condition, compared to the control groups, held less of an entity theory of personality, unstandardized *b* = −0.45, *SE* = 0.15, *t*(161) = 2.67 (covariate-adjusted *M*_incremental theory_ = 2.47, *M*_coping skills and no treatment_ = 2.92), *p* < .05, *d* = .47, controlling for baseline entity theory of personality. Thus, the incremental theory intervention successfully countered the belief that the type of person someone is in high school cannot be changed.

### Did the Intervention Improve Behavioral Responses to Peer Victimization or Exclusion?

#### Aggressive behavior

**Figure 2:**
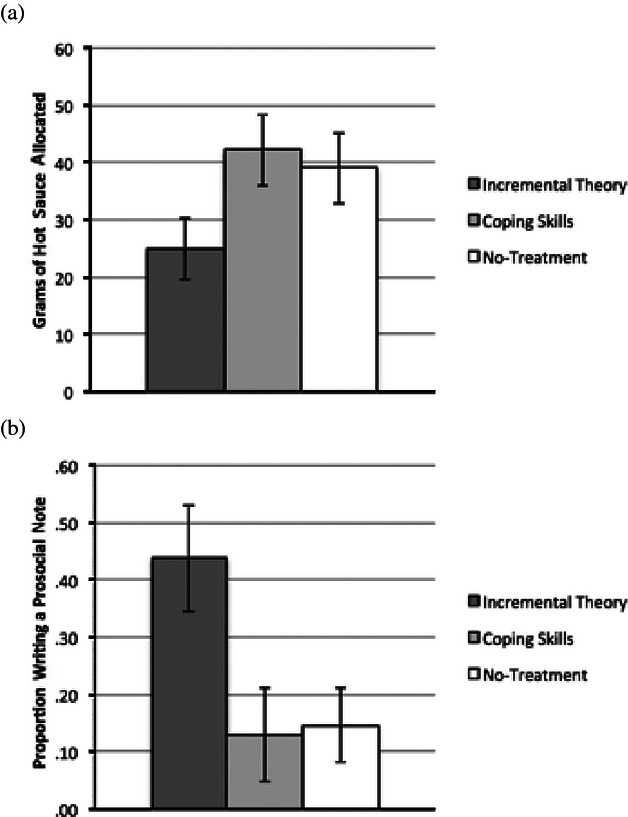
Aggressive and prosocial behavior. *Note*. Effect of intervention on behavioral responses to peer provocation, 1 month postintervention: (a) average grams of hot sauce allocated to Cyberball partner (covariate adjusted); (b) proportion who wrote a prosocial note to accompany hot sauce (covariate-adjusted). Error bars represent 1 *SE*. *p < .05.

Our major hypothesis was that behavioral aggression would be lower for the incremental theory group than the other groups 1-month postintervention. In line with this, students in the combined control groups assigned an average of 40.65 g of hot sauce—or about six spoonfuls—to the peer who had excluded them (*M*_coping skills_ = 42.18, *SD* = 36.95; *M*_no treatment_ = 39.03, *SD* = 34.14), indicating high levels of aggression on the basis of previous studies using this measure (Lieberman et al., 1999). Incremental theory group participants, however, assigned their partners almost 40% less hot sauce (*M*_incremental theory_ = 24.89 g, *SD* = 27.13), a significantly less aggressive response, *b =* −15.19, *SE* = 7.12, *t*(88) = −2.13, *p* < .05, *d* = .47 (see Figure 2a). Although the control groups did not differ from each other, individual comparisons revealed that the incremental theory group was significantly different from the coping skills control group (*p* < .05), and was marginally different (*p* = .09) from the no-treatment control group. This effect was equally strong for victims of peer aggression and nonvictims, Incremental Theory Condition × Victim Status interaction *b* = −5.46, *SE* = 16.42, *t*(88) = 0.33, *ns*, and for students who were thought of as aggressive or not aggressive by their peers, Incremental Theory Condition × Baseline Aggression interaction *b* = −0.83, *SE* = 3.78, *t*(88) = 0.22, *ns*.

#### Prosocial behavior

We then tested whether those in the incremental theory condition—who were taught that people could change—would act more prosocially after exclusion. They did. Fourteen percent of the control group participants wrote prosocial notes (*M*_coping skills_ = 13%; *M*_no treatment_ = 15%), whereas more than 3 times as many (44%) of those in the incremental theory condition did so, logistic regression unstandardized *b* = 1.68, *SE* = 0.55, *t*(88) = 3.07, *p* < .05, *d* = .86, a significant difference (see Figure 2b). The effect of the incremental theory intervention was significant compared to each of the control groups (*p*s < .05), and again, as predicted, the effect was not moderated by victim status or baseline levels of aggression. Overall then, the incremental theory intervention, with its message about the potential for change, not only decreased aggressive responses but also increased prosocial behavior following peer victimization.

### Was the Effect of the Intervention Noticeable to Teachers at the End of the School Year?

We next tested whether improvement in students' behavior would be evident to teachers 3 months postintervention. The incremental theory intervention participants received significantly more nominations from teachers for having reduced their conduct problems (*M*_incremental theory_ = 0.46 nominations per student, *SD* = 0.59) than did participants from both control groups (*M*_coping skills_ = 0.32, *SD* = 0.45; *M*_no treatment_ = 0.33, *SD* = 0.44), *b* = 0.14, *SE* = 0.07, *t*(226) = 2.05, *p* < .05, *d* = .29, which did not differ from each other. (These results were again confirmed when comparing the incremental theory with the combined control groups using a negative binomial test, incidence risk ratio = 1.46, *p* < .05, which some experts have suggested is an important additional test when analyzing count data; Cohen, Cohen, West, & Aiken, 2003.)

**Figure 3:**
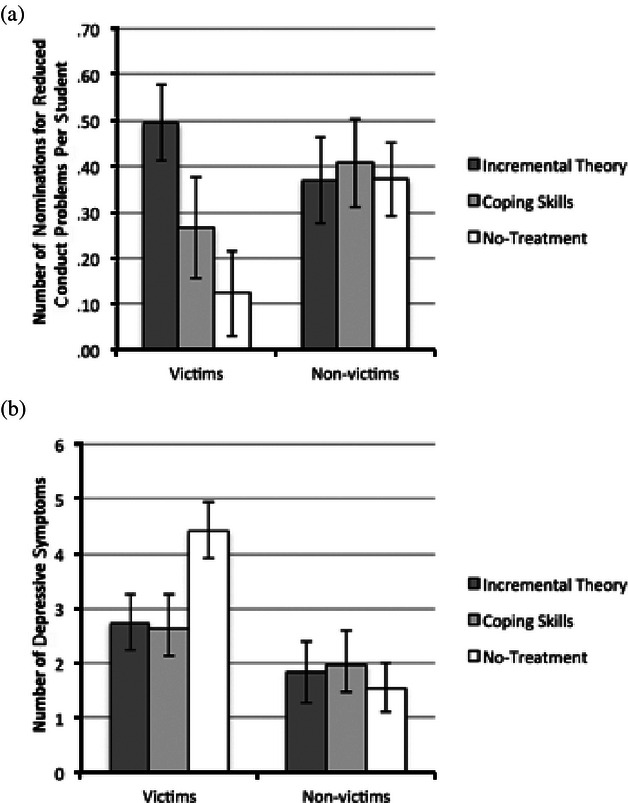
Conduct problems and depression. *Note*. (a) The effect of the incremental theory intervention on teacher nominations for reduced conduct problems for victims and nonvictims 3 months postintervention (covariate adjusted). (b) The effect of the incremental theory and coping skills interventions (compared to the no-treatment control) on reports of depressive symptoms for victims and nonvictims 2 weeks postintervention (covariate adjusted). Depressive symptoms reported on the 10-item short-form Childhood Depression Inventory (Kovacs, 1992), with potential scores ranging from 0 to 20. Error bars represent 1 *SE*. *p < .05.

Because the incremental theory intervention was designed to change the meaning of victimization or exclusion, we predicted that the intervention would change school behavior primarily for those who were experiencing victimization. We therefore asked whether the main effect of the incremental theory intervention was qualified by a significant Incremental Theory Condition × Victim Status interaction. It was, *b* = 0.33, *SE* = 0.16, *t*(161) = 2.09, *p* < .05. Specifically, among victims of peer aggression, teachers were more likely to nominate students who participated in the incremental theory intervention as showing reductions in conduct problems (victims: *M*_incremental theory_ = 0.49 nominations, *SD* = 0.60), compared to both control groups combined (victims: *M*_coping skills_ = 0.27, *SD* = 0.40; *M*_no treatment_ = 0.12, *SD* = 0.27), *b* = 0.30, *SE =* 0.10, *t*(73) = 2.99, *p* < .05, *d* = .66 (see Figure 3a). Among victims, the incremental theory group was significantly different from the no-treatment group (*p* < .05), and marginally different (*p* = .10) from the coping skills control group. Although nonvictims tended to show overall improvement (nonvictims: *M*_incremental theory_ = 0.37, *SD* = 0.58; *M*_coping skills_ = 0.41, *SD* = 0.46; *M*_no treatment_ = 0.37, *SD* = 0.48), there was no difference in nominations for nonvictims across the groups, *b* = −0.02, *SE =* 0.12, *t*(87) = −0.13, *ns*, *d* = .03. Thus, consistent with predictions, when considering nominations for improved conduct, the largest difference between the incremental theory group and the combined control groups was seen for those who could benefit most from a new mindset for coping with peer aggression—that is, those who reported peer victimization.

### Did the Intervention Reduce Depressive Symptoms Among Victims of Peer Aggression?

Replicating much past research, in the no-treatment control group, adolescents who were recent victims of peer aggression had depression scores that were nearly 3 times higher than those who were not currently victims (victims: *M*_no treatment_ = 4.42, *SD* = 3.86; nonvictims: *M*_no treatment_ = 1.55, *SD* = 2.12), *b* = 2.85, *SE* = 0.68, *t*(65) = 4.01, *p* < .05, *d* = .87 (see Figure 3b; cf. Klomek et al., 2007). For participants who received the incremental theory intervention *or* the coping skills intervention, however, the association between victimization and depressive symptoms was reduced. Indeed, the depression measure was the only outcome for which the coping skills intervention showed a significantly different pattern from the no-treatment group. In a regression predicting depressive symptoms, the Incremental Theory Condition × Victim Status and the Coping Skills Condition × Victim Status interactions were significant (victims: *M*_incremental theory_ = 2.75, *SD* = 4.05; *M*_coping skills_ = 2.63, *SD* = 3.22; nonvictims: *M*_incremental theory_ = 1.84, *SD* = 2.85; *M*_coping skills_ = 1.97, *SD* = 2.52), *b* = −1.96, *SE* = 0.98, *t*(158) = −2.00, *p* < .05; *b* = −2.16, *SE* = 1.07, *t*(158) = −2.02, *p* < .05, respectively (see Figure 3b). Among victims of peer aggression, incremental theory and coping skills participants reported significantly fewer depressive symptoms than no-treatment control participants, *p*s < .05, *d*s = .60 and .66, respectively (see also Figure 3b). Moreover, among incremental theory and coping skills participants, victimization no longer predicted greater depressive symptoms, *b* = 0.51, *SE* = 0.45, *t*(94) = 1.12, *ns*. Overall, this result suggests that both the incremental theory intervention and the coping skills intervention protected adolescents from some of the depressive symptoms that often accompany peer victimization.

### Did the Incremental Theory Intervention Decrease Absences and Tardies?

Previous research has shown that bullied adolescents are more frequently absent or tardy from class (e.g., Juvonen, Nishina, & Graham, 2000; Kochenderfer & Ladd, 1996). This may be due to a desire to escape victimization by missing a class or skipping school altogether or due to more doctor visits or sick days resulting from chronic stress (Gini & Pozzoli, 2009). We therefore conducted an exploratory analysis of school records to determine whether the incremental theory intervention—which was designed to make social exclusion or victimization seem less permanent and therefore might make school feel less hostile and stressful—might also reduce absences and tardies. In this analysis, we found that, overall, students who were taught an incremental theory were significantly less likely to be absent or tardy postintervention than those in both control groups combined, *b* = −0.43, *SE* = 0.21, *t*(229) = 2.04, *p* < .05, *d* = .27, controlling for preintervention attendance. This same effect was slightly (but not significantly) larger when considering only those students who reported being victims of peer aggression, *b* = −0.68, *SE* = 0.36, *t*(76) = 1.98, *p* < .05, *d* = .45. Thus, the new incremental mindset may have helped students to find school more hospitable or may have reduced physical symptoms resulting from the stress of school. It would be interesting in future research to test these potential mechanisms directly.

## Discussion

Our society continues to grapple with violence following victimization and exclusion among high school adolescents. In light of the past difficulty of reducing levels of aggression in this age group (e.g., Fossum et al., 2008; Merrell et al., 2008; Silvia et al., 2011; Smith et al., 2004; Vreeman & Carroll, 2007), it becomes important to advance developmental theories of the factors leading to retaliatory aggression in high school students and to test a new developmentally appropriate strategy to reduce them. Therefore, in the present research, we evaluated an intervention that taught an incremental theory—the idea that people have the potential to change—and found that it was successful in reducing levels of aggression, conduct problems, depression, and truancy for racially and socioeconomically diverse students.

More specifically, 1 month postintervention, we found that learning an incremental theory reduced aggressive retaliation after a controlled provocation (an experience of exclusion) by almost 40%, and increased prosocial behavior after the same event by over 300%, compared to the combined control groups of students who learned coping skills or who received no treatment. Notably, the effect of the incremental theory on behavioral responses to peer exclusion was not limited to victimized students, and appeared to work equally well for highly aggressive and less aggressive students. Hence, when students were put in a situation in which they all had to face an unexpected social challenge, those who learned the incremental theory behaved more resiliently, regardless of whether they normally encounter those challenges. This points to the broad theoretical importance of implicit theories as an influence on aggression.

Although students in general who were taught the incremental theory were prepared to respond less aggressively to an acute experience of victimization or exclusion, the effects of the incremental theory training on school conduct were mostly apparent for those who reported higher levels of victimization in school. Three months postintervention, victimized students who had received the incremental theory intervention were 2.5 times more likely to be nominated by their teachers for reductions in conduct problems (such as aggression and acting out in class), relative to victimized adolescents in the combined control groups. Taken together, our results suggest that an incremental theory may predispose students to behave resiliently when situations of exclusion or victimization arise. These situations arise more often for certain students, and therefore we might expect to see more widespread changes for those students.

Our findings can inform theories of how social cognitive development can influence adolescent aggression. Past research has suggested that adolescents show an increased belief in the fixed nature of transgressors' traits and behaviors (e.g., Killen et al., 2010). Relatedly, the early years of high school are a time of heighted social comparison, where one's social label (especially if it is seen as a fixed label) can be a source of pride or shame, and therefore a powerful influence on how one copes with peer conflict (e.g., Brown, Mory, & Kinney, 1994; Crosnoe, 2011; Eccles & Barber, 1999). Overall, adolescence was predicted to be a special period during which beliefs about the potential for people to change their personal characteristics could play a particularly important role in aggressive retaliation. It is possible that these beliefs, when unaddressed, may have prevented adolescents from profiting from past interventions that succeeded with children.

Peer victimization or exclusion, as we have noted, can also lead to depression and other internalizing symptoms, and previous correlational research has suggested that this is especially likely when children hold more of an entity theory (Rudolph, 2010). Our experimental study showed that an incremental theory intervention could buffer adolescents from the effects of peer victimization. When adolescents who reported higher levels of victimization were taught to see themselves and others as capable of change, they reported fewer depressive symptoms compared with adolescents who received no treatment. Interestingly, the group that learned coping skills and was taught to accentuate the positive also reported fewer depressive symptoms. However, they did not change their aggressive or prosocial reactions to exclusion, improve their classroom behavior relative to the no-treatment control group, or attend class more. Hence, perhaps an essential ingredient of effective aggression-prevention interventions in adolescence is an effort to change construals of oneself and the peers with whom one is in conflict, rather than just learn behavioral or emotional coping strategies.

Our conclusions are strengthened by our control group, which constituted a high standard against which to judge our incremental theory intervention. Students rated both workshops as equally enjoyable and informative. Further, the coping skills intervention was delivered by highly trained, experienced, and dynamic adult facilitators who incorporated many enjoyable and challenging team-building activities and who strongly believed in what they were teaching. The coping skills intervention directly taught students constructive ways of thinking and of responding to exclusion and victimization, while providing numerous opportunities for practicing and acting out those responses with scenarios. As testimony to the skillful and faithful implementation of the coping skills curriculum, this intervention had a significant positive effect on the depressive symptoms of victimized adolescents (though not on their aggression), conceptually replicating previous evaluations of the curriculum's beneficial effects on emotional coping (e.g., Frydenberg et al., 2004).

Our findings cannot speak to the efficacy of all coping or social skill-building interventions that address aggression among high school students. However, although the coping skills control group was chosen as a representative of an approach that has been widely used with children and adolescents, it was tailored to be parallel to the incremental workshop, and so this specific version had not been evaluated previously. In addition, the coping skills workshop was universal and brief—lasting only six class sessions—whereas many skill-building interventions are longer and target only at-risk students. Therefore, it is possible that a more extensive, targeted, and established skill-building control group would have produced better outcomes. However, this is not certain. As noted, long and targeted interventions increase the risk of “deviancy training” and stigma (Dishion & Tipsord, 2011; Dodge, Dishion, et al. 2006). Moreover, our coping skills control group intervention shared much in common with other coping skills interventions that are frequently evaluated in clinical trials but that produce no impact on aggression among adolescents (for a recent example, see Silvia et al., 2011). In addition, our universal incremental theory intervention was the same length as the coping skills intervention, and so length and the use of a universal sample cannot explain the differences in aggressive behavior.

### The Public's Lay Theories About Aggression and Victimization

On a broader level, the disappointing findings of many past interventions with adolescents may have fostered the idea among researchers and laypeople that aggressive tendencies are somewhat set in stone by mid to late adolescence. Such beliefs could have many repercussions. They could reduce the motivation to intervene in high school or to seek new interventions that might be more effective for adolescents. They may also lead to more punitive practices with adolescents rather than practices designed to educate or remediate. One example of a punitive practice is the zero tolerance policy, a policy of delivering severe consequences after a single instance of undesirable behavior. This not only failed to reduce conduct problems in school but also increased racial inequalities in discipline (Skiba et al., 2006). Moreover, a fixed belief about aggressive high school students could lead us to convey counterproductive (fixed) messages to adolescents who are the targets of aggression. For example, to make victims feel better, people may tell them that the bully is “just a bad person,” or that one should delight in a “Bully Beatdown.” Ironically, such messages—although designed to be comforting—may teach the same fixed belief about personality traits that have been shown to be a liability in the face of adversity (Yeager et al., 2011) and that we targeted for intervention in the present investigation. It therefore becomes important for researchers, the public, and educators to understand and communicate the potential malleability of aggression and victimization.

### Social-Cognitive Theories of Development

Our findings speak to more general theories of how children and adolescents come to construct their social worlds. Schemata (Piaget, 1932/1965), working models of relationships (Bowlby, 1958; see also Johnson, Dweck, & Chen, 2007), attributional styles (Nolen-Hoeksema, Girgus, & Seligman, 1992), or implicit theories (Dweck, 1999) can shape people's construals of and reactions to the environment to create consistent patterns of actions (Dweck, 2008; Olson & Dweck, 2008). Indeed, Block (1993) suggests that a central part of personality development is the formation of such “premise systems” on the basis of socialization experiences (see also Rothbart & Ahadi, 1994). For instance, the effect of domestic violence (Grych, Fincham, Jouriles, & McDonald, 2000), abuse (Feiring, Taska, & Lewis, 2002), or maternal depression (Garber, Keiley, & Martin, 2002) on children's subsequent adjustment is significantly influenced by whether children develop self-blaming views of these events. Similarly, the effect of harsh parenting and childhood rejection on later aggression is affected by whether children develop hostile patterns of social information processing (Pettit, Lansford, Malone, Dodge, & Bates, 2010). In the context of these theories and our study's findings, patterns of aggressive retaliation are better understood not as inborn traits or intractable habits, but rather as resulting in important ways from the social-cognitive frameworks that adolescents have developed. This more cognitive, constructivist account of aggression is more optimistic, to the extent that our research has shown that these frameworks are malleable (see also Yeager et al., 2011).

### Application to Educational Settings

Although it was important theoretically to show that implicit theories could have an impact on meaningful social behaviors among one sample of adolescents, we do not mean to suggest that reducing aggression, depression, and truancy in applied settings is a simple matter, requiring only that we tell students “people can change.” Importantly, we did not tell adolescents that people could change overnight, that change was easy or common, or that it was their responsibility to change others. The messages were piloted with students like those at our intervention site to make them nuanced, accurate, and believable, and statements from pilot students were quoted extensively throughout the workshop. Taking an intervention such as this to scale may well require customization, refinement, and additional evaluation to ensure that the incremental message hits its psychological mark (Yeager & Walton, 2011). This may impose logistical challenges and financial costs. Hence, our intervention is not intended as a “quick fix” to aggression.

Second, we agree with theories that characterize aggression as a complex behavior that is multiply-determined by ecological forces operating on many levels (Dodge, Coie, & Lynam, 2006; see also Bronfenbrenner, 1979). An implicit-theories intervention is certainly not a replacement for comprehensive initiatives such as improved school safety, better discipline policies, increased youth activities, community-building initiatives, parent education, or other efforts to promote positive youth development. Such initiatives are essential for reducing aggression, as research has documented (Dodge & Coie, 2006). However, our research does suggest that even in the absence of comprehensive reforms, a psychological intervention can make headway because it addresses problematic patterns of construals that may be preventing traditional interventions from being fully effective. For this reason, our intervention could serve as an important complement to—but not a replacement for—broader efforts.

### Extensions and Limitations

One extension of this research is to test whether implicit theories might also be a cause of bullying itself. It may be the case that some students bully others to validate themselves and their status, a motivation that may well be fostered by an entity view of the self. Indeed, adolescents who believe that there are fixed “winners” and “losers” may well wish to place themselves among the “winners” and use bullying as a tool for doing so. Thus, it may be interesting in future investigations to determine whether the present study's incremental theory intervention would reduce bullying.

Our research is not without limitations, however. First, our incremental theory intervention involved many different activities—some involving neuroscience, others involving peers, and still others involving discussions with teachers. As such, it is not clear which of these elements led to our effects. Thus, we are currently testing whether a briefer intervention can more precisely isolate the effect of the incremental theory treatment. Next, our sample had some attrition for the self-report measures, due to students declining to provide data on a given day or due to the frequent absences (on average, 25% of the school was absent on a given day). Even so, it is encouraging that for analyses in which there were no missing data (absences, teacher nominations for behavior), we observed a similar pattern of results. Last, we have proposed that an incremental theory intervention should make the most difference relative to social skills interventions during adolescence—and especially in high school—when fixed beliefs about traits and worries about negative social labels are at their height. Yet we think it will be important to test this directly.

### Conclusion

Victimization and exclusion are difficult experiences for any adolescent to cope with, whether they happen once in a while or every day. The present study showed that an intervention designed to teach adolescents that people have the potential for change could take the edge off these experiences and lead to less aggressive retaliation and more prosocial behavior. Moreover, this occurred in an age group and in a context believed by some to be relatively impervious to reform—an urban, diverse public high school with substantial levels of conflict. Going forward, our society would do well to incorporate a message of malleability into our conversations about the future prospects of both aggressive and victimized adolescents in this age group.

## Supporting Information

**Data S1.** Overview.

**Table S1.** Aggressive and Prosocial Behavior: Coefficients From Regressions Testing the Effects of the Incremental Theory Intervention on Behavioral Responses to a Peer Provocation 1 Month Postintervention.

**Table S2.** Mean Levels of Behavioral Responses to a Peer Provocation.

**Table S3.** Reduction in Conduct Problems: Coefficients From OLS Regressions Testing Effects of the Intervention and Victim Status on Number of Teacher Nominations for Reduced Conduct Problems per Student 3 Months Postintervention.

**Table S4.** Mean Number of Teacher Nominations (from 0 to 2) per Student for Reduced Conduct Problems by Victim Status and Experimental Condition.

**Table S5.** Depressive Symptoms: Coefficients From OLS Regressions Testing Effects of Incremental and Coping Skills Interventions, and Victim Status, on Depressive Symptoms 2 Weeks Postintervention.

**Table S6.** Mean Number of Depressive Symptoms Reported (by Victimization Status and Experimental Condition).

**Figure S1.** Examples of Notes Written to Accompany Hot Sauce Allocation.
