# Supplementary material for: An Implicit Theories of Personality Intervention Reduces Adolescent Aggression in Response to Victimization and Exclusion
Source: Child Dev. 2012 Oct 25;84(3):970–88. doi: 10.1111/cdev.12003 (PMC3660787; doi:10.1111/cdev.12003)

**ONLINE SUPPLEMENT FOR:**

**An Implicit Theories Intervention Reduces Adolescent Aggression in Response to Victimization and Exclusion**

**Overview**

In this supplement, we include the following materials:

- Additional detail about the measures of aggression (peer-nominated) and conduct problems (teacher-nominated).
- Detailed descriptions of the procedures used to measure behavioral responses to victimization / exclusion.
- Tests of the effectiveness of random assignment.
- Tests of the equality of victimized students across experimental conditions.
- Tests of the equality of the Incremental Theory and Coping Skills interventions in terms of knowledge of the brain and enjoyment of the sessions.
- Evidence for the criterion validity of the Cyberball exclusion and hot sauce measure relative to additional behavioral measures of aggression.
- Supplemental analyses of the teacher nominations data using a different analytic method.
- Tables presenting the regression models and covariate adjusted means for the measures of behavioral responses to victimization (hot sauce and notes; Tables S1 and S2), teacher nominations for improvements in conduct (Tables S3 and S4), and depression (Tables S5 and S6).
- A figure presenting copies of the notes written to hot sauce partners (Figure S1).

**Method**

**Tests of Equality of Interventions**

At the end of every two sessions of the workshops, participants in both the Incremental Theory and Coping Skills interventions were asked to respond to two questions about the sessions, “How much do you feel like you enjoyed the past two days?” and “How much do you feel like you learned in the last two days?” each on a fully-labeled five-point scale (1 = *Not at all*, 5 = *A great deal*). Additionally, at the end of the first two days, students in both the Incremental and Coping Skills control group completed the same five-question quiz on the parts and functions of neurons and the brain.

**Peer Nominations for Aggression**

Due to a clerical error, only 1/3 of the students in the No-Treatment control group were listed on the pre-survey and could be rated for baseline levels of aggression. Aggression ratings for the remaining students in that group were estimated using the multiple imputation software Amelia II, King, Honaker, Joseph, & Scheve, 2001). The original design called for a post-intervention peer assessment of aggression. However, in debriefing interviews following the pre-survey we found that this peer nomination procedure caused a great deal of stress and anger among students. We therefore used a teacher report of reduction in conduct problems as a post-test measure.

**Teacher Nominations for Reductions in Conduct Problems**

All of the boys were listed on one screen, and all of the girls were listed on a separate screen, randomized for each teacher. Boys’ and girls’ names were presented on separate pages in order to prevent the under-estimation of girls’ aggression that might occur if they were compared to boys’ aggression, which may be more likely to be direct and observable by teachers (Pelligrini, 2011). The order of presentation of the list of boys and the list of girls was counterbalanced across teachers. Each teacher could nominate each student a maximum of two times (once for decreased misbehavior and once for decreased aggression). Two students in the Incremental Theory group were nominated many times, and such outliers in count data can skew results in favor of finding treatment effects (Schneider, Tahk, & Krosnick, 2007). To be conservative, we set the nominations for these two outliers to be equal to 2, the highest value for the other participants.

**Methods for the Behavioral Response to Victimization and Exclusion Task**

**Set-up**. When students arrived they were seated in a private cubicle and were told by Experimenter 1 that they would be participating in two experiments: a video game experiment and a food-tasting experiment. They were also told that they would be interacting via computer with students in their grade who were in cubicles in another room. They were reminded of the confidentiality of their data and the voluntary nature of their participation.

Next, Experimenter 1 introduced them to Experimenter 2, who said he was acting as a food researcher. He asked students to complete a food inventory (that had their subject number and not their name on it). On the inventory they indicated on a 21-point scale the degree to which they liked or disliked various types of foods, including “spicy or hot” foods. After students completed the inventory, Experimenter 2 collected the sheets and left, stating that he was taking them to the next room to share them with the other students.

**Peer exclusion or victimization.** Once Experimenter 2 had left, Experimenter 1 told students that they would now participate in his experiment, which was on “how visualization affects your memory.” They were told that they would play a virtual ball-tossing game called “Cyberball,” and that the computer would randomly pair them with two partners from the other room. In reality, the game was controlled by the computer (Williams & Jarvis, 2006). Following standard practice, the two figures representing the other players were programmed to throw the ball twice to the participant initially and then only to each other – not the participant - for a few minutes. Students were told that their task was to visualize the game as it was happening, and in particular to imagine what the other players were thinking and what was going through their minds. Extensive testing with various populations has found that Cyberball is experienced as a meaningful (temporary) incident of peer ostracism (Williams & Jarvis, 2006; Williams, 2009). Importantly, Cyberball does not have lasting negative effects on students because, as we have noted, with debriefing and a final good experience with the Cyberball game, they quickly recover from the online exclusion (Williams & Jarvis, 2006), making it ideal for our purposes.

After being excluded by partners in Cyberball, students were given a “memory task.” First, they wrote the subject numbers of the other players in Cyberball (the same for all students). Next, for each of the two other players, they were asked to write an answer to this question, “Do you feel like this subject threw the ball to you very few times or very many times?” (Response options: *Not at all, A few times, A lot of times, Very many times*). This activity was designed to lead them to recognize the subject numbers on the subsequent task. It also served as a manipulation check on the Cyberball task. One hundred percent of students said they received the ball “not at all” or “a few times.”

**Aggressive behavior (hot sauce allocation) and prosocial behavior (notes to peer)**. Once students’ memory responses were collected, Experimenter 1 re-introduced Experimenter 2, who told students that he would now be continuing the study of food preferences. Experimenter 2 then gave students private boxes so that they could assign a food sample to their partner without the experimenter seeing. They were informed that, because the class was running out of time, their partner in the study was going to be one of the same subjects they had just played Cyberball with. Next, students were given their partner’s food preference inventory, formatted exactly like the inventory they themselves had completed earlier. It was ostensibly filled out by “Subject 7,” and all of the foods were rated as being liked, except for “spicy or hot,” which was rated only a 3 out of 21. To draw students’ attention to the negative rating of the spicy or hot food, the rating-scale label “extreme disliking” for that food was circled three times. Students were instructed to read the taste inventory carefully before opening their box. Research assistants checked that each participant had looked at it before Experimenter 2 gave the instruction to open the box and begin the task.

All boxes were prepared identically. Inside was an instruction sheet, a mason jar filled halfway with hot sauce and marked “HOT” in red ink (all jars were filled to the same point), a Styrofoam cup and a Styrofoam lid, two spoons (one to try the food and one to assign it), a Kleenex to clean their hands, and a blank piece of paper to send their partner a message. The instruction sheet indicated that they had been randomly assigned to allocate spicy and hot food.

The sheet instructed them to first try a small amount of the food and next to decide how much they wanted their partner to try. Then they were instructed to put that amount of food in the Styrofoam cup provided in the box. The sheet also said that the partner would have to consume the entire food sample that they provided.

The instruction sheet next asked them to write a message to their partner, and told them that they could anonymously write whatever they wanted to.

Finally, students placed all the materials back in their boxes, which were collected by Experimenter 2, put on a cart, and taken out of the room to be weighed. Each cup was immediately weighed twice by trained assistants and the average weight was used in the analysis.

**Debriefing**. Once Experimenter 2 left the room, Experimenter 1 said that the experiment was almost over, but that they would play Cyberball one last time. In this second game, to make students feel included, all students received the ball as many times as the other two players, following standard practice for “inclusion” (Williams, 2009). Because some students might have regretted assigning large amounts of hot sauce, we told all students that we were running late so their partners would not have time to eat the food sample they had prepared.

A full debriefing was postponed until the end of the day because both the researchers and the school principal agreed that, if debriefed, students would be unlikely to refrain from talking to other students about the study. The students in earlier periods were requested not to share information about the procedures with other students and, if asked, should say to other students “If I told you it would ruin all the fun.” When students were debriefed, they were reassured that they were not actually excluded or ignored, that they were playing with a computer that was programmed to not throw them the ball, and, again, that no one ate the hot sauce sample they prepared. They were told that these procedures were necessary to ensure that students acted naturally, and that we could not learn how to help students who are excluded and ignored unless we could observe their real behavior. They were then told that their participation was extremely helpful in teaching us about how to help students, and that many teachers and parents will do a better job because they participated. Students were encouraged to ask questions and these questions led to fruitful discussions with the whole group. They were thanked again and told they could speak with the researcher further if they so desired. Many students did so, and of those who did, all said that they were happy to participate and contribute to an important topic.

**Results**

**Effectiveness of Random Assignment**

To examine the effectiveness of random assignment, one-way ANOVAs testing for differences among the three conditions revealed no differences at baseline in terms of implicit theories of personality, peer nominations for aggressive behavior, depressive symptoms, absences or tardies, or prior grades in four core subjects (all *p*s > .10). Additionally, χ^2^(2) tests revealed that there were no differences in terms of sex, grade level, suspensions for fighting or defiance, or race and ethnicity (all *p*s > .10). Finally, the sub-samples of participants who did not participate in the dependent measures did not vary from those who did participate in terms of these same variables across conditions, except for the grade level variable. (Among the participants who did not complete the post-survey, those in the incremental condition were more likely to be in the 10^th^ grade, *p* < .05; however, among those who completed the survey, there was no difference in grade level across conditions, *n.s.*). Therefore, as noted, we controlled for grade level in all analyses.

**Analysis of Victimization** **Levels**

We considered the possibility that the different experimental messages altered the reporting of victimization for students with different characteristics. If this were the case, then the predictors of victimization status might be different across conditions. We therefore conducted a series of analyses to test whether the associations between victimization and other baseline variables (GPA in four core subjects, sex, grade level, entity theory of personality, aggression nominations, and depressive symptoms) significantly varied across conditions. We conducted nine separate regressions predicting the baseline variables. In these analyses, the Incremental Theory condition × Victimization status interactions were in every case non-significant (*p*s > .10), suggesting that the characteristics of victims did not vary across conditions.

**Tests of Equality of Interventions**

Students in both treatment groups said on average that they had a “moderate amount” to “a lot” of fun (*M* = 3.72 out of 5), and the amount of enjoyment did not differ between the two groups (*M*_Incremental Theory_ = 3.73, *SD*= 0.90; *M*_Coping Skills_ = 3.72, *SD*= 1.00), *t*(332) = 0.39, *n.s*. (note that on some days some class sessions did not have time to complete these measures). On the test of brain facts, it appeared that the coping skills group learned somewhat more about the brain, (*M*_Incremental Theory_ = 3.89 correct answers out of 5, *SD*= 0.75; *M*_Coping Skills_ = 4.30 correct answers, *SD*= 0.51), *t*(165) = 3.66, *p* < .05. Thus, if the incremental theory group was different from the coping skills control group, it would not be because they learned more about the brain.

**Validity of Measures of Aggressive Behavior**

We also assessed the validity of our measure of aggressive behavior. To do so, we first examined whether students who were suspended for fighting or conduct problems before the intervention allocated more hot sauce in response to an experience of peer victimization or exclusion (in the Cyberball game). They did. Students suspended for fighting or conduct problems assigned more than twice as many grams of hot sauce, *b* = 36.56, *SE* = 17.01, *t*(91) = 2.15, (*M*_Suspended_ = 71.52 grams, *M*_Not suspended_ = 34.96 grams), *p* < .05, *d* = 1.08. In addition, students who wrote prosocial notes assigned less than half as much hot sauce to their partners than those who did not, unstandardized *b* = -21.80, *SE* = 7.75, *t*(91) = 2.81, (*M*_Prosocial_ = 17.33 grams, *M* _Not prosocial_ = 39.13 grams), *p* < .05, *d* = .58. Last, students’ nominations for reduced conduct problems three months post-intervention were significantly negatively correlated with the grams of hot sauce they allocated one month post-intervention, polychoric *r*(91) = -.28, *p* < .05. Overall, this evidence is consistent with many past studies that have documented the validity of behavioral aggression measured in experimental tasks (Anderson & Bushman, 1997).

**Supplementary Analysis of Teacher Nominations for Reduced Conduct Problems**

Fifty-seven percent of study participants were not nominated for reduced conduct problems, and some statisticians have noted that traditional linear models have the potential to inflate t-statistics when there is a large number of zeros in the distribution of the dependent variable (J. Cohen, P. Cohen, West, & Aiken, 2003; Long, 1995; see also Schneider et al., 2007). We therefore re-conducted our analyses of the teacher ratings using a negative binomial test. Statisticians have recommended the negative binomial model for the analysis of count data, as long as observations were obtained independently (Cohen et al., 2003; Long, 1995), as was the case in our study, which used independent teacher nominations. We found that this alternative model did not yield different results: we found a significant effect of an incremental theory in the full sample (Incidence risk ratio = 1.46, *p* = .037), and a larger effect within the sub-sample of victims (Incidence risk ratio = 2.60, *p* = .002)

**Supplemental References**

Anderson, C.A., & Bushman, B.J. (1997). External validity of "trivial" experiments: The case of laboratory aggression. *Review of General Psychology, 1*, 19-41.

Blackwell, L., Trzesniewski, K., & Dweck, C. S. (2007). Implicit theories of intelligence predict achievement across an adolescent transition: A longitudinal study and an intervention. *Child Development, 78*, 246-263.

Cohen, J., Cohen, P., West, S. G., & Aiken, L. S. (2003). *Applied multiple regression / correlation analysis for the behavioral sciences* (3rd ed.). Mahwah, NJ: Lawrence Erlbaum Associates Inc.

Frydenberg, E. (2010). *Think positive: A course for developing coping skills in adolescents.* London: Continuum.

Long, J. S. (1995). *Regression models for categorical and limited dependent variables.* Thousand Oaks, CA: Sage Publications.

Pelligrini, A.D. (2011). “In the eye of the beholder”: Sex bias in observations and ratings in children’s aggression. *Educational Researcher, 40,* 281-286.

Schneider, D., Tahk, A., & Krosnick, J. A. (2007). Reconsidering the impact of behavior prediction questions on illegal drug use: The importance of using proper analytic methods. *Social Influence, 2*, 178-196.

Williams, K. D. (2009). Ostracism: A temporal need-threat model. In M. Zanna (Ed.), *Advances in Experimental Social Psychology, 41*, (pp. 279-314). NY: Academic Press.

Williams, K. D., & Jarvis, B. (2006). Cyberball: A program for use in research on ostracism and interpersonal acceptance. *Behavior Research Methods, Instruments, and Computers, 38,* 174-180*.*

**Table S1.** Aggressive and Prosocial Behavior: Coefficients from Regressions Testing the Effects of the Incremental Theory Intervention on Behavioral Responses to a Peer Provocation One Month Post-Intervention.

|  | Regression predicting amount of aggression (hot sauce allocation)^a^ | | | |  | Regression predicting proportion behaving prosocially (notes)^b^ | | | | |
| --- | --- | --- | --- | --- | --- | --- | --- | --- | --- | --- |
| Predictor | *b* | *SE* | *t* | *p* |  | *b* | *SE* | *t* | *p* |  |
| Condition (1 = Incremental; 0 = Coping and No-Treatment) | -15.19 | 7.12 | -2.13 | .033 | * | 1.68 | 0.55 | 3.07 | .002 | * |
| Grade level (1 = 10^th^; 0 = 9^th^) | 0.53 | 6.94 | 0.08 | .940 |  | 0.16 | 0.55 | 0.30 | .766 |  |
| Sex (1 = Male; 0 = Female) | 4.46 | 7.07 | 0.63 | .528 |  | -0.88 | 0.57 | -1.55 | .121 |  |
| Baseline aggression | -0.84 | 2.00 | -0.42 | .676 |  | 0.05 | 0.15 | 0.32 | .748 |  |
| Intercept | 43.13 | 8.61 | 5.01 | .000 | * | -2.10 | 0.73 | -2.88 | .004 | * |
| *N* | 92 |  |  |  |  | 92 |  |  |  |  |

Note: *b* = un-standardized regression coefficients; *SE* = standard error. ^a^ OLS regression; ^b^ Logistic regression. * *p* < .05.

**Table S2.** Mean Levels of Behavioral Responses to a Peer Provocation.

| Condition | *M* | 95% CI | | | *d* |  |
| --- | --- | --- | --- | --- | --- | --- |
| Aggression (hot sauce allocation in grams) |  |  |  |  |  |  |
| Incremental Theory | 24.89 | (14.84 | , | 34.79) |  |  |
| Coping Skills | 42.18 | (29.62 | , | 54.38) |  |  |
| No-Treatment | 39.03 | (27.15 | , | 51.77) |  |  |
| *Incremental vs. Coping and No-treatment* |  |  |  |  | .47 | * |
| Prosocial notes (proportion) |  |  |  |  |  |  |
| Incremental Theory | .44 | (.27 | , | .63) |  |  |
| Coping Skills | .13 | (.04 | , | .29) |  |  |
| No-Treatment | .15 | (.05 | , | .30) |  |  |
| *Incremental vs. Coping and No-treatment* |  |  |  |  | .86 | * |

Note: *M* = estimated mean; CI = confidence interval; *d* = Cohen’s *d* standardized difference between means. Models adjust for sex, grade level, and baseline levels of aggression (peer nominations). * *p* < .05.

**Table S3.** Reduction in Conduct Problems: Coefficients from OLS Regressions Testing Effects of the Intervention and Victim Status on Number of Teacher Nominations For Reduced Conduct Problems Per Student Three Months Post-Intervention.

|  | Model 1 | | | |  | Model 2 | | | | |
| --- | --- | --- | --- | --- | --- | --- | --- | --- | --- | --- |
| Predictor | *b* | *SE* | *t* | *p* |  | *b* | *SE* | *t* | *p* |  |
| Condition (1 = Incremental; 0 = Coping and No-Treatment) | .14 | .07 | 2.05 | .040 | * | -.01 | .11 | -0.14 | .892 |  |
| Victim status (1 = Victim; 0 = Non-victim) |  |  |  |  |  | -.20 | .09 | -2.19 | .028 | * |
| Condition × Victim status |  |  |  |  |  | .33 | .16 | 2.09 | .036 | * |
| Grade level (1 = 10^th^; 0 = 9^th^) | .01 | .07 | 0.11 | .912 |  | .06 | .07 | 0.77 | .441 |  |
| Sex (1 = Male; 0 = Female) | .03 | .07 | 0.48 | .635 |  | .05 | .07 | 0.72 | .472 |  |
| Baseline aggression | .04 | .02 | 2.19 | .029 | * | .05 | .02 | 2.15 | .033 | * |
| Intercept | .17 | .08 | 2.17 | .031 | * | .20 | .09 | 2.19 | .028 | * |
| *n* | 230 |  |  |  |  | 167 |  |  |  |  |

Note: *b* = un-standardized regression coefficients; *SE* = standard error. * *p* < .05.

**Table S4.** Mean Number of Teacher Nominations (from 0-2) Per Student for Reduced Conduct Problems by Victim Status and Experimental Condition.

| Condition | *M* | 95% CI | | | *d* |  |
| --- | --- | --- | --- | --- | --- | --- |
| All participants |  |  |  |  |  |  |
| Incremental Theory | .46 | (.36 | , | .57) |  |  |
| Coping Skills | .32 | (.20 | , | .43) |  |  |
| No-Treatment | .33 | (.21 | , | .44) |  |  |
| *Incremental vs. Coping and No-treatment* | |  |  |  | .29 | * |
| By victimization status |  |  |  |  |  |  |
| Victims |  |  |  |  |  |  |
| Incremental Theory | .49 | (.33 | , | .66) |  |  |
| Coping Skills | .27 | (.04 | , | .47) |  |  |
| No-Treatment | .12 | (-.06 | , | .31) |  |  |
| *Incremental vs. Coping and No-treatment* | |  |  |  | .66 | * |
| Non-victims |  |  |  |  |  |  |
| Incremental Theory | .37 | (.18 | , | .56) |  |  |
| Coping Skills | .41 | (.22 | , | .59) |  |  |
| No-Treatment | .37 | (.21 | , | .53) |  |  |
| *Incremental vs. Coping and No-treatment* | |  |  |  | .03 | *ns* |

Note*: M* = estimated mean; CI = confidence interval; *d* = Cohen’s *d* standardized difference between means. Models adjust for sex, grade level, and baseline levels of aggression (peer nominations). * *p* < .05.

**Table S5.**  Depressive Symptoms: Coefficients from OLS Regressions Testing Effects of Incremental and Coping Skills Interventions, and Victim Status, on Depressive Symptoms Two Weeks Post-Intervention.

|  | Model 1 | | | |  | Model 2 | | | | |
| --- | --- | --- | --- | --- | --- | --- | --- | --- | --- | --- |
| Predictor | *b* | *SE* | *t* | *p* |  | *b* | *SE* | *t* | *p* |  |
| Incremental condition (1 = Incremental; 0 = No-Treatment) | -.48 | .52 | -0.92 | .357 |  | .26 | .68 | 0.38 | .707 |  |
| Coping Skills condition (1 = Coping; 0 = No-Treatment) | -.42 | .55 | -0.76 | .448 |  | .38 | .69 | 0.55 | .581 |  |
| Victim status (1 = Victim; 0 = Non-victim) |  |  |  |  |  | 2.85 | .68 | 4.17 | .000 | * |
| Incremental × Victim status |  |  |  |  |  | -1.96 | .98 | -2.00 | .046 | * |
| Coping × Victim status |  |  |  |  |  | -2.16 | 1.07 | -2.02 | .043 | * |
| Baseline depression | .64 | .08 | 7.99 | .000 | * | .58 | .08 | 7.16 | .000 | * |
| Grade level (1 = 10^th^; 0 = 9^th^) | .40 | .44 | 0.93 | .354 |  | .29 | .42 | 0.70 | .484 |  |
| Sex (1 = Male; 0 = Female) | -.11 | .44 | -0.24 | .810 |  | -.25 | .43 | -0.58 | .564 |  |
| Baseline aggression | .11 | .15 | 0.75 | .454 |  | .07 | .13 | 0.54 | .592 |  |
| Intercept | .90 | .63 | 1.44 | .150 |  | .11 | .46 | 0.23 | .814 |  |
| *n* | 170 |  |  |  |  | 167 |  |  |  |  |

Note: Depressive symptoms reported on the 10-item short-form *Childhood Depression Inventory* (Kovacs, 1992), with potential scores ranging from 0-20. *b* = un-standardized regression coefficients; *SE* = standard error. * *p* < .05.

**Table S6.** Mean Number of Depressive Symptoms Reported (By Victimization Status and Experimental Condition).

| Condition | *M* | 95% CI | | | *d* |  |
| --- | --- | --- | --- | --- | --- | --- |
| Victims |  |  |  |  |  |  |
| Incremental Theory | 2.75 | (1.76 | , | 3.73) |  |  |
| Coping Skills | 2.63 | (1.38 | , | 3.85) |  |  |
| No-Treatment | 4.42 | (3.36 | , | 5.41) |  |  |
| *Coping vs. No-Treatment* |  |  |  |  | -.66 | * |
| *Incremental vs. No-Treatment* |  |  |  |  | -.60 | * |
| Non-victims |  |  |  |  |  |  |
| Incremental Theory | 1.84 | (0.78 | , | 2.93) |  |  |
| Coping Skills | 1.97 | (0.93 | , | 3.02) |  |  |
| No-Treatment | 1.55 | (0.74 | , | 2.41) |  |  |
| *Coping vs. No-Treatment* |  |  |  |  | .14 | *ns* |
| *Incremental vs. No-Treatment* |  |  |  |  | .10 | *ns* |

Note: Depressive symptoms reported on the 10-item short-form *Childhood Depression Inventory* (Kovacs, 1992), with potential scores ranging from 0-20. M = estimated mean; CI = confidence interval; *d* = Cohen’s *d* standardized difference between means. Models adjust for sex, grade level, baseline levels of aggression (peer nominations), and baseline depressive symptoms. * *p* < .05

**Figure S1.** Examples of Notes Written to Accompany Hot Sauce Allocation.

(a)


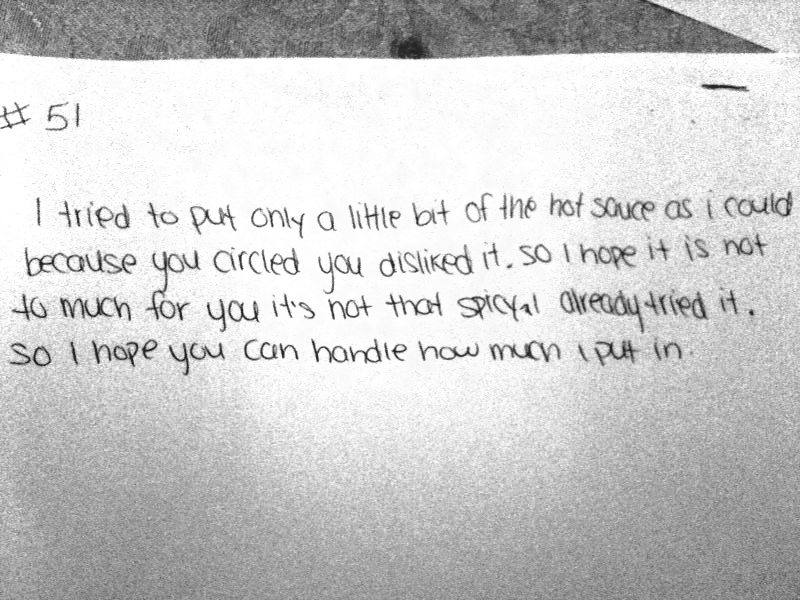


(b)


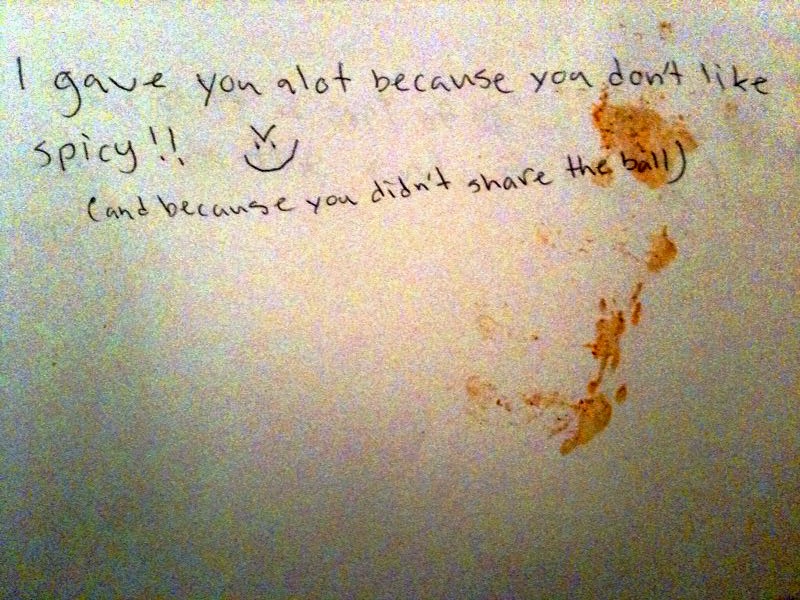

Supplement: Supplementary file 1 [file cdev0084-0970-SD1.doc]
